# Optimizing Anticoagulation Strategies in Patients With Atrial Fibrillation and Valvular Heart Disease: A Comprehensive Evidence-Based Review

**DOI:** 10.7759/cureus.81319

**Published:** 2025-03-27

**Authors:** Dharani S Deiveegan, Mohamed Salahie, Muhammad Subhan, Sulman Ismail, Muhammad Abdullah Khan, Darshankumar M Raval, Usama Abbas, Beyla Betsy Baiju, Husam K Abuasaker, Ruqiya Bibi

**Affiliations:** 1 Internal Medicine, The Tamil Nadu Dr. M. G. R. Medical University, Tiruchirappalli, IND; 2 Pediatrics, Upstate University Hospital, Syracuse, USA; 3 Medicine, Allama Iqbal Medical College, Lahore, PAK; 4 Internal Medicine, Akhtar Saeed Medical and Dental College, Lahore, PAK; 5 General Medicine, King's Mill Hospital, Sutton-in-Ashfield, GBR; 6 Internal Medicine, Sir Sayajirao General (SSG) Hospital, Maharaja Sayajirao (MS) University, Vadodara, IND; 7 Physiology, University College of Medicine and Dentistry, University of Lahore, Lahore, PAK; 8 Medicine and Surgery, Tbilisi State Medical University, Tbilisi, GEO; 9 Internal Medicine, Beni-Suef University Hospital, Beni-Suef, EGY; 10 Internal Medicine, University of Sinnar, Sinnar, EGY

**Keywords:** atrial fibrillation (af), direct acting oral anticoagulant, echocardiography, heart failure, international normalized ratio (inr), low-molecular weight heparin, mitral stenosis (ms), rivaroxaban dosing, valvular heart disease, warfarin

## Abstract

Atrial fibrillation (AF), the most common sustained cardiac arrhythmia, significantly increases the risk of thromboembolism and stroke. Its coexistence with valvular heart disease (VHD) further complicates management due to elevated risks of thromboembolism, bleeding, and mortality. This review explores the pathophysiology of AF and its interaction with VHD, focusing on diagnostic tools like echocardiography and risk stratification scores such as CHA2DS2-VASc and HAS-BLED. Vitamin K antagonists (VKAs) remain the cornerstone of anticoagulation therapy in high-risk VHD populations, particularly in patients with mechanical heart valves or moderate-to-severe mitral stenosis (MS). VKAs have demonstrated proven efficacy in reducing thromboembolic events in these subgroups, supported by decades of clinical evidence. However, their use requires frequent international normalized ratio (INR) monitoring and is associated with higher bleeding risks, posing challenges in long-term management. Despite these limitations, VKAs are indispensable in these populations due to the lack of robust evidence supporting the safety and efficacy of direct oral anticoagulants (DOACs) in these high-risk groups. Ongoing clinical trials, such as the RIVER trial, aim to evaluate the role of DOACs in VHD. However, current guidelines continue to recommend VKAs as the standard of care for these patients. In contrast, DOACs offer significant advantages in non-valvular AF and selected VHD populations. Their predictable pharmacokinetics, fewer dietary restrictions, and lower risks of intracranial hemorrhage make them a preferred choice for many patients. Landmark trials and meta-analyses, including RE-LY, ROCKET-AF, and ARISTOTLE, have demonstrated the safety and efficacy of DOACs in non-valvular AF and certain VHD subgroups. However, DOACs are contraindicated in high-risk VHD populations, such as those with mechanical valves or moderate-to-severe MS, due to insufficient evidence and potential risks of thromboembolic events. Evolving guidelines from leading societies emphasize individualized approaches and collaborative decision-making in anticoagulation therapy. While DOACs are preferred for most AF patients, VKAs remain essential for high-risk VHD patients. Future advancements, such as factor XIa inhibitors, hold promise for improving outcomes and safety in these complex populations. This review provides a comprehensive framework for clinicians to navigate the complexities of anticoagulation in AF and VHD, ensuring evidence-based, patient-centered care.

## Introduction and background

Atrial fibrillation (AF) is one of the most prevalent cardiac arrhythmias, characterized by abnormal electrical activity in the atrial chambers leading to irregular, rapid beats often manifested as tachyarrhythmia [1]. Valvular heart disease (VHD) refers to abnormalities affecting the four cardiac valves: mitral, aortic, tricuspid, and pulmonary valves [2,3]. In the United States, degenerative valve disease is the most common form of VHD, whereas rheumatic heart disease accounts for most valvular pathology worldwide [1,2]. The coexistence of AF and VHD is frequently observed in clinical practice and presents significant management challenges due to the increased risk of thromboembolic events, heart failure, and stroke [3-7].

The interplay between AF and VHD extends beyond their coexistence, with each condition exacerbating the progression of the other. VHD, particularly mitral stenosis (MS) and mitral regurgitation (MR), contributes to left atrial enlargement and elevated left atrial pressure, which promote electrical and structural remodeling, key drivers of AF initiation and maintenance [8,9]. Conversely, AF exacerbates VHD by increasing turbulent blood flow across diseased valves, accelerating valvular degeneration, and worsening hemodynamic instability [10]. This bidirectional relationship creates a vicious cycle that amplifies the risk of thromboembolism, stroke, and heart failure, underscoring the need for integrated management strategies [9,10].

Globally, approximately 60 million individuals are affected by AF, with a steadily increasing prevalence [3]. In the United States, the prevalence of AF is projected to rise from 5.2 million in 2010 to 12.1 million by 2030 [4]. Men are more likely to develop AF, with estimated prevalence rates of 596.2 per 100,000 men versus 373.1 per 100,000 women [6]. While aging significantly contributes to AF, growing evidence suggests that genetic predisposition, structural remodeling, and ion channel dysfunction also play key roles in disease progression [7,8]. The pathophysiology of AF includes electrical remodeling, calcium handling abnormalities, and autonomic dysregulation, all of which are exacerbated by coexisting VHD [8]. Clinically, AF symptoms range from asymptomatic cases to severe presentations with palpitations, fatigue, and dyspnea [9]. VHD can further aggravate these symptoms, potentially leading to heart failure and other complications [10].

Despite the availability of well-established clinical guidelines, some argue that AF management in the presence of VHD does not require further exploration. However, clinical guidelines often generalize treatment strategies, and individualized patient care is essential due to the heterogeneous nature of AF and VHD [11]. Moreover, anticoagulation therapy, a cornerstone of AF management, becomes particularly complex when VHD is present, requiring careful consideration of stroke risk versus bleeding complications [11,12]. This review responds to concerns regarding the adequacy of current guidelines by highlighting emerging challenges, novel treatment strategies, and areas for further investigation.

Technological advancements in imaging, artificial intelligence (AI)-driven diagnostics, and personalized medicine hold promise for addressing the complexities of AF and VHD more effectively than previously anticipated [12]. For example, AI algorithms can enhance risk stratification by integrating clinical, imaging, and genetic data to accurately predict thromboembolic and bleeding risks, thereby guiding personalized anticoagulation strategies [13]. Similarly, advancements in transcatheter interventions, such as transcatheter aortic valve replacement (TAVR) and minimally invasive mitral valve repair, are transforming the therapeutic landscape for patients with concomitant AF and VHD [14]. These innovations improve procedural outcomes and influence anticoagulation decisions by reducing the need for long-term anticoagulation in certain patient subgroups.

Unquestionably, the aging population contributes to the increased occurrence of AF, but it is essential to acknowledge that enhanced diagnostic tools and medical awareness also play a role. Additionally, the disease's increasing prevalence is driven by environmental and lifestyle factors such as obesity, hypertension, and metabolic syndrome [15]. Similarly, VHD shares several lifestyle-related risk factors, including hypertension, diabetes, and smoking, which contribute to valvular calcification and degeneration [16]. Thus, attributing AF and VHD prevalence solely to aging oversimplifies the conditions' intrinsic complexities and highlights the need for comprehensive risk factor management [15].

Previous research has primarily focused on anticoagulation strategies, stroke prevention, and procedural interventions in AF and VHD [15-17]. However, significant gaps remain in refining risk stratification models, optimizing patient-specific treatment protocols, and assessing the long-term efficacy of novel therapeutic approaches. For instance, the safety and efficacy of direct oral anticoagulants (DOACs) in high-risk VHD populations, such as those with mechanical heart valves or moderate-to-severe MS, remain inadequately studied [18-21]. Additionally, the role of factor XIa inhibitors and other emerging anticoagulants in AF and VHD requires further investigation through large-scale randomized controlled trials (RCTs) and real-world data analysis. Future efforts should emphasize interdisciplinary collaboration between cardiologists, electrophysiologists, and biomedical engineers to address these gaps and improve patient outcomes.

## Review

Association between atrial fibrillation and valvular heart disease

**Figure 1:**
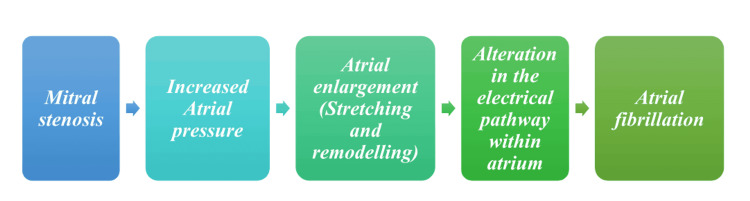
Pathophysiological pathway of mitral stenosis leading to atrial fibrillation Image Credit: Muhammad Subhan

AF, which often occurs independently in patients with preexisting VHD, should be distinguished from AF caused directly by valvular pathologies; the latter type is sometimes called Valvular AF. Valvular AF is a subtype of AF that is directly caused by valvular pathologies such as MS and artificial heart valves. This distinction is essential as it helps identify the underlying cause of AF, which can guide treatment decisions and improve patient outcomes. Unfortunately, due to an absence of explicit distinction, this term hasn't gained much acceptance clinically [2,3]. Figure 1 shows AF's pathogenesis stages due to MS [1-3].

Valve replacement surgeries play a significant role in the occurrence of AF [3,4]. A study conducted at the National Heart Institute in Malaysia focused on the incidence and predictors of postoperative AF (POAF) in patients undergoing coronary artery bypass grafting (CABG) surgery [5].

This retrospective, single-center, cross-sectional study involved 637 patients who underwent CABG. The analysis revealed that 28.7% of the patients developed POAF, with a mean onset of 45 ± 33 hours postoperatively. The study identified advancing age, the Indian population, a history of chronic kidney disease, left ventricular ejection fraction, and beta-blocker treatment as significant predictors of POAF [5].

The outcomes for patients who developed POAF were notably worse, with higher mortality rates, increased incidence of stroke, and other postoperative complications. These patients also had significantly more extended stays in the ICU, high-dependency unit, and hospital, with higher ICU readmissions and reintubations. The study concluded that the incidence of POAF in Malaysia is comparable to that in Western countries and that POAF is associated with poorer outcomes and increased healthcare costs. Implementing strategies to reduce POAF could improve surgical outcomes and decrease resource utilization [5]. This implies that doctors may be able to lower the prevalence of POAF and enhance patient outcomes by recognizing and controlling these variables.

Another study conducted a meta-analysis and systematic review of RCTs emphasizing the connection between AF and mortality after gastrointestinal (GI) procedures. Despite a comprehensive search across multiple databases, the trial showed no statistically significant difference in mortality between AF and non-AF groups, with a mortality OR of 1.03 and a 95% CI of 0.24 to 4.41. However, significant heterogeneity was noted across the trials, suggesting that the results should be depicted cautiously [6].

The study concluded that, although no significant link was found between AF and mortality after GI surgery, the complexity of the relationship and conflicting data from other research highlight the need for further extensive and diverse studies to better understand the role of AF in postoperative mortality related to GI procedures [6].

POAF is often observed after surgery to correct MR, even in those without a history [5,6]. This POAF is associated with increased morbidity and may be affected by factors like postoperative inflammation, scar tissue formation, and underlying conditions. Studies indicate it occurs in an estimated percentage of cardiac surgery patients, with rates depending on surgery type and patient characteristics [5-7].

AF can often happen as a side effect of conservatively managed MR, as its presence can lead to atrial enlargement and pressure increases, creating an ideal environment for its development. Addressing MR may help alleviate symptoms, while rhythm control strategies may be utilized to optimize patient care outcomes [7,8].

Effective treatment and management of AF and VHD depend on understanding their connection. Appropriate therapeutic approaches and better patient outcomes may inform treatment choices and maximize results by differentiating between AF brought on by valvular diseases and AF that occurs on its own in individuals with VHD [9,10].

Efficacy and safety of direct oral anticoagulants versus warfarin in nonvalvular atrial fibrillation: insights from major clinical trials

Not all patients with AF require anticoagulation therapy, as the decision to initiate treatment depends on individual risk factors for stroke and bleeding [11-15].

Before the advent of DOACs (apixaban, dabigatran, edoxaban, and rivaroxaban), warfarin was the mainstay for treating moderate- and high-risk AF patients [21-23]. However, its use was often hindered by a narrow therapeutic window (international normalized ratio (INR) 2-3), frequent monitoring requirements, susceptibility to drug interactions (primarily via CYP2C9), dietary restrictions, and an inferior safety profile [24].

DOACs were developed to overcome these limitations and are now recommended as first-line therapy for most patients with AF, except those with moderate to severe MS or mechanical heart valves [25-29]. All four pivotal clinical trials comparing DOACs with warfarin demonstrated superiority or noninferiority for preventing stroke or systemic embolism, with generally reduced risks of major bleeding, particularly intracranial hemorrhage (ICH) [30-35].

A study by Vinogradova et al. observed significantly decreased risks of major bleeding among patients treated with apixaban, dabigatran (110 mg twice daily), and edoxaban (30 mg or 60 mg daily) compared to warfarin. However, GI bleeding risks were higher with dabigatran 150 mg twice daily, edoxaban 60 mg once daily, and rivaroxaban, while apixaban did not significantly increase this risk [36].

Despite their advantages, DOACs are often more expensive than warfarin, which remains a cost-effective alternative for patients with affordability issues. Real-world prescribing trends show that DOACs are increasingly preferred due to their convenience and safety profile, but warfarin continues to be widely used in low-resource settings [35,36].

The PACIFIC-AF Trial evaluated the comparative efficacy of asundexian, a factor XIa inhibitor, against apixaban and found that asundexian significantly reduced major bleeding while providing comparable stroke prevention efficacy [37]. This highlights the potential of factor XIa inhibitors to address the unmet need for anticoagulants that minimize bleeding risks without compromising efficacy.

Other trials, such as PROGRESSIVE-AF, RAPID, NOVA, and EHANCE-AF, have also focused on optimizing anticoagulation strategies in AF, with findings supporting DOACs as more efficacious alternatives to warfarin [38]. A recent systematic review and meta-analysis compared the safety and efficacy of DOACs against warfarin in AF patients with a history of falls, finding that DOACs were associated with significantly fewer ICHs and ischemic strokes [39-43].

The evolution of anticoagulation therapy in AF has been marked by the development of more precise risk stratification tools and safer, more effective treatments like DOACs. While DOACs have largely replaced warfarin as first-line therapy, emerging therapies such as factor XIa inhibitors offer the potential for further improvements in patient outcomes. Ongoing research and updated guidelines will continue to refine anticoagulation strategies, ensuring that patients receive personalized, evidence-based care.

The advantages and disadvantages of vitamin K antagonist (VKA) and DOAC are displayed in Table 1 [16-20].

**Table 1:** Benefits and disadvantages of VKA and DOAC in a clinical setting INR: international normalized ratio, PCC: prothrombin complex concentrate, VKA: vitamin K antagonist, DOAC: direct oral anticoagulant [16-20]

Category	VKA	DOAC
Drug name	Warfarin	Dabigatran (direct thrombin inhibitor), rivaroxaban, apixaban, and edoxaban (direct factor Xa inhibitors)
Benefits	More cost-effective and affordable, easy monitoring of INR and adjusting doses in high-risk cases, easily reversible with vitamin K and PCC, sufficient clinical trials and studies supporting its usage, more prevalent and familiar dynamics, and can be used in pregnancy and other high-risk conditions	Predictable pharmacokinetics, rapid onset of action, no need for routine lab or INR monitoring, fixed dosages, reduced bleeding risk, and lower risk of thromboembolism
Disadvantages	Varies in drug response due to polymorphism, various drug interactions, slow onset, requires rigorous monitoring, reversal agents may take time, requires dietary restrictions, and slow clearance	Expensive, not easily reversible, limited availability of reversal agents, difficult to monitor rigorously, limited number of clinical trials and studies, not extensively explored in high-risk thrombosis and bleeding conditions

Comprehensive risk stratification and anticoagulation strategies in atrial fibrillation management

Not all patients with AF require anticoagulation therapy, as treatment depends on individual risk factors for stroke and bleeding [29]. Various risk stratification tools are available to guide therapy in patients with AF; commonly used scoring tools include the CHADS2 and CHA2DS2-VASc scores for assessing stroke risk and HAS-BLED scores to gauge bleeding risk [29,30]. CHADS2 was initially developed as an initial scoring system to detect stroke risk for AF patients [30]. Over time, however, other scoring systems, such as CHA2DS2-VASc, have expanded it by including additional risk factors and refining age categories; both scoring systems range from 0 to 6, with higher scores signifying more significant risks in each case [29,30]. Table 2 depicts a detailed explanation of the CHA2DS2-VASc Score [28-30].

**Table 2:** Utilization of the CHA2DS2-VASc score Sc: sex category, LV: left ventricular, HCM: hypertrophic cardiomyopathy, TIA: transient ischemic attack, CAD: coronary artery disease, PAD: peripheral arterial disease, MI: myocardial infarction, HF: heart failure [28-30]

CHA2DS2-VASc score	Risk factors and definitions	Points awarded	Comment
C	Congestive heart failure	1	Clinical HF, or objective evidence of moderate to severe LV dysfunction, or HCM
H	Hypertension	1	Or on antihypertensive therapy
A	Age 75 years or older	2	-
D	Diabetes mellitus	1	Treatment with oral hypoglycemic drugs and/or insulin or fasting blood glucose >125 mg/dL (7 mmol/L)
S	Stroke	2	Previous stroke, TIA, or thromboembolism
V	Vascular disease	1	Angiographically significant CAD, previous MI, PAD, or aortic plaque
A	Age 65−74 years	1	-
Sc	Sex category (female)	1	-
Maximum score		9	

Once a score is analyzed with tools like CHA2DS2-VASc, anticoagulant selection and dosage are determined according to current guidelines [28]. The 2020 European Society of Cardiology (ESC) guidelines for AF management recommend oral anticoagulation (OAC) for stroke prevention in AF patients with a CHA2DS2-VASc score of ≥2 in men or ≥3 in women. Additionally, OAC should be considered in patients with a CHA2DS2-VASc score of 1 in men or 2 in women, with treatment decisions individualized based on net clinical benefit and patient preferences [28-30].

However, the 2023 American College of Cardiology (ACC) guidelines have updated these recommendations. The ACC now advises that OAC is recommended for patients with a CHA2DS2-VASc score of ≥2, regardless of sex. This represents a shift from previous guidelines that differentiated recommendations based on sex. The 2023 guidelines from the ACC, American Heart Association (AHA), American College of Chest Physicians (ACCP), and Heart Rhythm Society (HRS) emphasize the importance of comprehensive risk stratification in managing AF [31].

In addition to the widely used CHA2DS2-VASc score, these guidelines recommend the use of ATRIA (Anticoagulation and Risk Factors in Atrial Fibrillation) and GARFIELD-AF (Global Anticoagulant Registry in the FIELD-Atrial Fibrillation) scores for a more nuanced assessment of stroke and bleeding risks. The ATRIA score is particularly valuable for predicting stroke and bleeding risks by incorporating factors such as age, prior stroke, and renal function, offering a more detailed risk assessment than CHA2DS2-VASc [31]. Studies have shown that ATRIA better identifies low-risk patients for stroke, aiding clinicians in making informed decisions regarding anticoagulation therapy [31-33].

Similarly, the GARFIELD-AF score is an integrated tool that predicts mortality, stroke, and bleeding risks in AF patients and has been validated across diverse populations, allowing for improved risk stratification and personalized treatment plans [31-33]. These scores support enhanced clinical decision-making by identifying patients who may benefit from anticoagulation while minimizing the risk of bleeding. Integrating ATRIA and GARFIELD-AF scores into clinical practice fosters a more individualized approach to managing AF, improving the ability to predict and mitigate associated risks [32,33].

The HAS-BLED score (hypertension, abnormal renal/liver function, stroke, bleeding history or predisposition, labile INR, elderly (age ≥65 years), drugs/alcohol concomitantly) has become a widely used tool for assessing bleeding risk. It is considered superior to other bleeding prediction scores such as HEMORR₂HAGES (hepatic or renal disease, ethanol abuse, malignancy, older age (≥75 years), reduced platelet count or function, hypertension (uncontrolled), anemia, genetic factors, excessive fall risk, and stroke). The ATRIA score, which includes factors like anemia, renal disease, age ≥75 years, and prior bleeding, also contributes to assessing bleeding risks. However, HAS-BLED's more comprehensive consideration of clinical factors makes it a preferred tool for predicting bleeding risks in patients undergoing anticoagulation therapy [34].

The ORBIT (Outcomes Registry for Better Informed Treatment) bleeding score assesses major bleeding risk using markers like hemoglobin levels, prior bleeding history, renal impairment, age, and antiplatelet use. The ABC (Age, Biomarkers, and Clinical History) stroke and bleeding scores also incorporate biomarkers such as NT-proBNP and troponins for more personalized risk prediction [35]. These tools aid clinicians in optimizing anticoagulation therapy by balancing stroke prevention with bleeding risks [31-35].

Regardless of risk stratification systems and guidelines, patients at moderate and high risk are prescribed OACs like warfarin (VKA) [31-33]. Before DOACs became widely available, warfarin was the mainstay for treating moderate- and high-risk AF patients. However, its use was often hindered by factors such as a narrow therapeutic window determined by INRs, frequent monitoring requirements, susceptibility to drug interactions (primarily via CYP2C9), dietary restrictions, and an inferior clinical safety profile. Studies indicate that targeting an INR between 2 and 3 is optimal, with increased risks evident once this threshold is exceeded [35].

DOACs were developed to overcome the drawbacks associated with warfarin and are currently recommended as first-line therapy in patients with AF, except for those with moderate to severe MS or mechanical heart valves [31-35]. All four pivotal clinical trials comparing DOACs (apixaban, dabigatran, edoxaban, and rivaroxaban) with warfarin demonstrated superiority or noninferiority in preventing stroke or systemic embolism among AF patients, except those with moderate to severe MS or mechanical heart valves [16-20,31-34].

A study by Vinogradova et al. observed significantly decreased risks of major bleeding among groups treated with apixaban, dabigatran (110 mg twice daily), and edoxaban (30 mg or 60 mg daily) compared with warfarin. There were no significant differences in major bleeding risk between the dabigatran 150 mg twice daily group and the rivaroxaban group compared to warfarin. However, all DOAC groups demonstrated significantly reduced ICH risks compared to warfarin. In contrast, GI bleeding risks were higher among patients taking dabigatran 150 mg twice daily, edoxaban 60 mg once daily, and rivaroxaban compared to warfarin users. In contrast, the apixaban group did not significantly increase this risk [36].

Because some AF patients struggle to afford DOACs, warfarin remains a viable OAC due to its lower cost [35,36]. In the PACIFIC-AF Trial, Jonathan P. Piccini evaluated the comparative efficacy of asundexian, a Factor XIa inhibitor, against apixaban regarding major bleeding reduction while maintaining similar efficacy for stroke prevention. Asundexian demonstrated significantly less major bleeding while providing comparable stroke protection [37].

The PROGRESSIVE-AF, RAPID, NOVA, and EHANCE-AF trials all focused on optimizing anticoagulation strategies in AF, with findings supporting DOACs as more efficacious alternatives to warfarin for treating atherosclerotic cardiovascular disease [38]. A recent systematic review and meta-analysis compared the safety and efficacy of DOACs against warfarin in AF patients with a history of falls, finding that DOACs were associated with significantly fewer ICHs and ischemic strokes [39-43].

DOACs (apixaban, dabigatran, edoxaban, and rivaroxaban) have demonstrated superior or non-inferior efficacy compared with warfarin for preventing stroke and systemic embolism while generally reducing the risk of major bleeding, including ICH [39]. DOACs do not require frequent INR monitoring and have fewer dietary restrictions, making them a more convenient option for many patients [43].

Anticoagulants targeting Factor XIa may provide promising strategies for lowering stroke risk while simultaneously mitigating bleeding complications [42-44]. Recent clinical trials, drug comparisons, and updated guidelines offer a comprehensive view of anticoagulation strategies in AF. While DOACs remain preferable over warfarin due to their improved safety profile and convenience, ongoing research and the development of novel anticoagulants, such as Factor XIa inhibitors, promise to further enhance patient outcomes [44-48].

Mitral annular calcification progression, stroke risk, and evolving treatment strategies in valvular heart disease

Lee et al. conducted a study aiming to elucidate factors associated with mitral annular calcification (MAC) progression and its clinical consequences. A retrospective study analyzed 138 patients with mild or moderate MAC diagnosed by transthoracic echocardiography and followed up 18 to 36 months later. MAC progression, defined as deteriorating hemodynamic or structural profiles of more than 1 grade, was noted in 31.2% of patients. Systolic blood pressure, pulse pressure, MAC angle (angle of the mitral annulus calcification) and transmitral mean diastolic pressure gradient (MDPG) are significant predictors of MAC progression. Interestingly, pulse pressure and MDPG were noteworthy individually prominent variables that predicted poorer clinical outcomes, while pulse pressure via chronic arterial stiffness and hemodynamic burden may be a potent driver. Patients with progressive MAC had higher rates of all-cause mortality, heart failure hospitalization, and ischemic stroke [45].

The distinction between VHD alone and VHD combined with AF is important due to different risks for stroke [45]. VHD, particularly MS and mechanical valve replacements, presents a high thromboembolic risk without AF [46]. Nonetheless, this risk is markedly furthered with pre-existing AF due to better left atrial contractility and blood stasis resulting in additives to embolic potential. According to studies, VHD patients with AF experience more cardiovascular events, bleeding complications, and mortality than patients with VHD alone [45,46]. This means that strategies of following VHD patients and their treatment should be adapted to this additive risk, with a more nuanced approach to anticoagulation and intervention [46,47].

The incidence of postoperative stroke was 4.3% in a study of 417 patients undergoing surgical valve replacement by Alwaqfi et al. Increasing stroke susceptibility was associated with numerous factors, such as long cardiopulmonary bypass time, aortic cross-clamp time >90 minutes, previous stroke, diabetes mellitus, and MAC. There was a significant increase in stroke risk for combined procedures: aortic valve replacement (AVR) with mitral valve replacement (MVR) or CABG with AVR and MVR (OR=10.74, CI: 2.65-43.44, p<0.001 and OR=11.66, CI: 1.02-132.70, p=0.048, respectively). This risk (ORs) appears to be markedly higher in cases where patients undergo the additional simultaneous procedure (MVR in conjunction with AVR) due to longer operative times, greater cardiopulmonary bypass period, and hemodynamic needs. The risk for stroke was also higher with internal carotid artery stenosis and prolonged inotropic support (OR=3.04, CI: 1.13-8.12, p=0.026). Extended ICU stay and in-hospital mortality were associated with postoperative strokes [46].

The current trends in treatment for VHD underscore the increasing role of the transcatheter therapy approach. New RCTs have emerged, mainly assessing surgical versus transcatheter approaches for AS, demonstrating that TAVR is non-inferior and compares favorably to SAVR in a select population. TAVR's benefit is greatest in high-risk surgical patients and the elderly due to its minimally invasive nature and limited perioperative morbidity [47]. The current ESC and European Association for Cardio-Thoracic Surgery (EACTS) Guidelines for VHD highlight accurate diagnosis, timely intervention, and individual risk assessment [45,46]. Such guidelines recommend TAVR for patients at prohibitive surgical risk and SAVR for younger patients with long-term durability concerns [47].

In the context of anticoagulation management, both VKAs and non-VKA oral anticoagulants (NOACs) have been used in patients with VHD. Still, there has been a significant transition toward NOAC use in this patient population, excluding patients with mechanical valves [47,48]. The safety profiles of NOACs used in AF patients are considerably more favorable than that of warfarin. NOACs are well tolerated and provide a lower risk of bleeding than warfarin in these patients with specific non-rheumatic VHD [47]. But mechanical valve recipients still need VKAs due to the absence of any evidence supporting the efficacy of NOAC for this subgroup [47,48].

Although progress has been made, there are still some important areas that remain to be explored. The long-term outcomes of TAVR in younger patients, the best strategies for prevention of stroke in MAC, and the best path of anticoagulation for VHD patients with AF may be areas of continued investigation. Further refinements to VHD management may emerge from studies that integrate precision medicine approaches to optimize therapeutic interventions.

Comparative analysis of oral anticoagulant therapy in patients with atrial fibrillation and valvular heart disease

**Figure 2:**
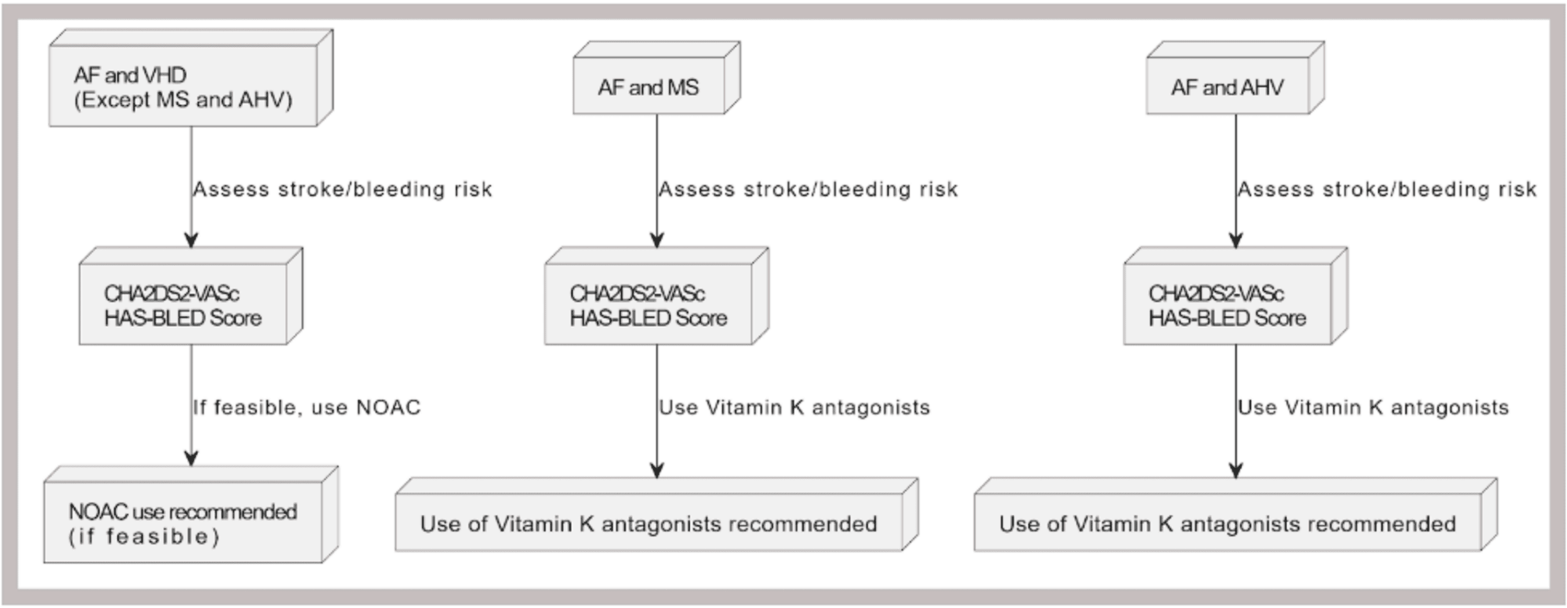
Basic recommendation for using anticoagulants in patients with AF and VHD AF: atrial fibrillation, VHD: valvular heart disease, NOAC: non-vitamin K antagonist oral anticoagulants, MS: mitral stenosis, AHV: aortic heart valve Image Credit: Ruqiya Bibi

According to the 2024 AHA/ACC guidelines for managing VHD (excluding MS and bioprosthetic valves), the decision to use anticoagulation therapy, whether a VKA or a NOAC, for the prevention of thromboembolic events should be made collaboratively with the patient. This decision should be guided by the CHA2DS2-VASc score to assess stroke risk accurately. Studies and clinical trials involving patients with VHD or VHD with AF (excluding high-risk VHD) who use NOACs have produced more positive outcomes in terms of reduced systemic embolization (SSE), myocardial infarction (MI), and ICH when compared with warfarin use [48]. Figure 2 shows the basic outlines to start anticoagulants in specific conditions.

The ENGAGE AF-TIMI 48 trial evaluated the use of edoxaban in patients with AF, including those with VHD (excluding MS and bioprosthetic valves), and found that the presence of VHD did not impact the efficacy or safety of higher-dose edoxaban compared to warfarin. However, it is important to note that patients with high-risk VHD, such as MS or mechanical heart valves, were excluded from this study [49]. A meta-analysis by Zhang et al. specifically evaluated the efficacy and safety of DOACs versus warfarin in patients with AF and significant MS, a subgroup often excluded from NOAC trials. The study, which included data from 835,520 patients across multiple RCTs and observational studies, found that DOACs had similar risks of stroke or SEE (HR: 0.51; 95% CI: 0.09-2.96), all-cause death (HR: 0.81; 95% CI: 0.35-1.87), and major or clinically relevant non-major bleeding (HR: 0.57; 95% CI: 0.24-1.39) compared to warfarin. These findings suggest that DOACs are comparable to warfarin in efficacy and safety for patients with AF and significant MS, challenging the traditional preference for VKAs in this population [50].

The European Heart Rhythm Association (EHRA), ESC, HRS, Asia-Pacific Heart Rhythm Society (APHRS), and Latin American Society of Electrophysiology and Cardiac Stimulation (SOLAECE) joint consensus document introduced a new classification system for oral anticoagulant therapy in AF patients, categorizing VHD into Type 1 and Type 2 [51]. EHRA Type 1 VHD includes patients who require VKA therapy, such as those with mechanical heart valves or severe MS, while EHRA Type 2 VHD encompasses patients who may benefit from either VKAs or NOACs [51,52]. A 2019 comparative study evaluated the predictive value of the CHA2DS2-VASc and HAS-BLED scores in patients with concurrent AF and VHD (including severe MS and aortic valve disease). The study found that both scores had modest predictive values for thromboembolism and bleeding risks, with CHA2DS2-VASc showing a c-index of 0.62 (95% CI: 0.50-0.70) for thromboembolism and HAS-BLED showing a c-index of 0.59 (95% CI: 0.53-0.65) for bleeding [52]. These results underscore the need for improved risk stratification tools in this patient population [52,53].

Clinical trials on NOAC use in patients with AF and bioprosthetic valves have been limited but increasingly supportive of NOACs as a viable alternative to VKAs [53-60]. A meta-analysis by Caldeira et al. reviewed data from three trials involving 280 patients with bioprosthetic valves and found that NOACs were associated with similar risks of thromboembolism (HR: 0.65; 95% CI: 0.20-2.08) and major bleeding (HR: 0.94; 95% CI: 0.28-3.18) compared to VKAs [53]. Similarly, a meta-analysis by Yokoyama et al. involving 6,405 patients with bioprosthetic valves and AF found that DOACs were associated with a significantly lower risk of major bleeding (HR: 0.66; 95% CI: 0.48-0.89, p=0.006) and a similar risk of stroke or SEE (HR: 0.72; 95% CI: 0.44-1.17, p=0.18) compared to VKAs [58]. These findings suggest that DOACs may offer a safer alternative to VKAs in patients with bioprosthetic valves, though the risk of bleeding remains a concern [51-60].

A retrospective cohort study by Yadlapati et al. evaluated NOAC use in 73 patients with bioprosthetic valve implantation and AF, reporting no ischemic strokes (0.0%) and only one possible transient ischemic attack (1.4%) [61-63]. However, minor bleeding events occurred in 8.2% of patients, and major bleeding events were observed in 6.9% [63]. These results highlight the efficacy of NOACs in reducing thromboembolic events but also underscore the need for careful monitoring of bleeding risks [63-66]. The use of NOACs in patients with mechanical heart valves or high-risk VHD, such as MS, remains controversial. The RE-ALIGN trial, which evaluated dabigatran in patients with mechanical heart valves, was terminated early due to increased rates of stroke (5% in the dabigatran group vs. 0% in the warfarin group) and major bleeding (4% vs. 2%, respectively). The adverse outcomes were attributed to dosing concerns and patient selection, as dabigatran’s pharmacokinetics may not be suitable for mechanical valves' high shear stress and thrombogenic environment [67]. This trial underscores the importance of cautious patient selection and dosing when considering NOACs in high-risk VHD populations. In contrast, a study by Koertke et al. evaluated low-dose INR self-management in patients with mechanical heart valve prostheses and found no significant increase in thromboembolic events compared to conventional-dose INR management (0.37% vs. 0.19% per patient-year, p=0.79). However, bleeding events requiring hospitalization were slightly higher in the low-dose group (1.52% vs. 1.42%, p=0.69). While the difference was not statistically significant, the study highlights the need for long-term validation of low-dose INR strategies in this population [64].

NOACs are comparable to VKAs in efficacy and safety for patients with AF and VHD, excluding high-risk subgroups such as MS and mechanical heart valves [65]. NOACs are a viable alternative to VKAs in patients with bioprosthetic valves, offering similar thromboembolic protection with a potentially lower risk of major bleeding [66,67]. The use of NOACs in patients with mechanical heart valves or severe MS remains contraindicated due to increased risks of stroke and bleeding, as demonstrated by the RE-ALIGN trial [67,68]. The CHA2DS2-VASc and HAS-BLED scores have modest predictive value in patients with AF and VHD, highlighting the need for improved risk assessment tools. While NOACs are effective in reducing thromboembolic events, they are associated with a notable risk of bleeding, particularly in patients with bioprosthetic valves or those undergoing low-dose INR management [68]. Further large-scale studies are needed to validate these findings and refine risk stratification and dosing strategies for NOACs in diverse VHD populations.

Novel oral anticoagulants in patients with AF and moderate to severe mitral stenosis

The latest guidelines from the ACC, AHA, ESC, and EACTS unanimously advise against the use of NOACs in patients with AF and moderate to severe MS [20-23].

The 2020 ACC/AHA guidelines specifically recommend warfarin for these patients due to insufficient data supporting the safety and efficacy of NOACs in this population [23-26,61]. These guidelines emphasize that valvular AF, which includes moderate to severe MS, requires long-term anticoagulation with warfarin [23-25]. Similarly, the 2021 ESC/EACTS guidelines recommend warfarin as the preferred anticoagulant for patients with clinically significant MS, citing a high risk of thromboembolism and the absence of robust clinical trial data supporting NOACs for these patients [26]. According to these significant cardiovascular authorities, NOACs are contraindicated for this condition [23,24].

Recent studies, such as the RISE MS and X-VeRT trials, provide valuable insights into managing anticoagulation in severe MS [68,69]. The RISE MS study, though a smaller trial with only 40 patients, suggested that rivaroxaban could be an alternative to warfarin for anticoagulation in severe MS, with similar rates of silent cerebral ischemia (13.3% for rivaroxaban vs. 17.6% for warfarin). However, the small sample size limits these findings' statistical power and generalizability, making it difficult to draw definitive conclusions. Larger RCTs are needed to validate these results [68].

The X-VeRT trial (2016), involving 1,399 patients, primarily studied elective cardioversion strategies for AF but included a subgroup analysis of patients with MS. The trial found that rivaroxaban, while non-inferior to warfarin in preventing stroke and systemic embolism, was associated with a significantly higher rate of GI bleeding (1.5% vs. 0.5% for warfarin) in patients with MS. Importantly, this subgroup analysis was exploratory and not predefined, which limits its reliability. Additionally, rivaroxaban’s lack of routine monitoring makes it less reliable in this population, where precise anticoagulation control is crucial [69].

In contrast, warfarin, despite requiring frequent INR monitoring, offers more predictable anticoagulation, which is especially important in severe MS to prevent both thromboembolic events and bleeding risks. Warfarin’s well-documented risk of intracranial bleeding, while significant, is outweighed by its established safety profile and the ability to closely monitor and adjust therapy. The contraindication of NOACs in moderate to severe MS is not solely based on the lack of data but also specific mechanistic concerns [69]. Severe MS is associated with altered hemodynamics, increased left atrial pressure, and a higher burden of thrombus formation, which may require more predictable and tightly controlled anticoagulation [23,24].

Warfarin’s mechanism of action, which targets multiple clotting factors, may be better suited to managing the hypercoagulable state in MS than NOACs, which target specific factors (e.g., factor Xa or thrombin) [23,24]. Additionally, the lack of routine monitoring for NOACs makes it difficult to ensure therapeutic efficacy and safety in this high-risk population [69].

The meta-analysis by Liang et al. provides the most comprehensive evidence supporting NOAC use in AF patients with Type 2 VHD, demonstrating a significant reduction in stroke and systemic embolism risk compared to VKAs. However, this study lacks detailed subgroup analysis for individual NOACs, limiting its application in selecting specific agents for different patient populations [41]. Importantly, moderate to severe MS is classified as EHRA Type 1 VHD, for which NOACs are contraindicated. In contrast, Type 2 VHD includes conditions like bioprosthetic valves and non-severe MS, where NOACs may be safe and effective [23,24,41]. This distinction is critical for clinical decision-making [22,23].

The study by Yoon et al. utilizes real-world data to emulate RCTs, reinforcing NOAC efficacy in venous thromboembolism (VTE) management. However, as an observational study, it is susceptible to biases such as residual confounding and selection bias, which limit its ability to establish causality [42].

Similarly, the systematic review by Adhikari et al. suggests that DOACs reduce the risk of major bleeding without increasing thromboembolism risk in AF patients with bioprosthetic valves. Still, it does not differentiate among individual NOACs, making it difficult to determine the safest option in this subgroup [43]. The population-based cohort study by Dawwas et al. provides critical insight into intra-class differences among NOACs, demonstrating that apixaban is more effective and safer than rivaroxaban in AF patients with VHD [44].

This finding is consistent with other studies highlighting apixaban’s favorable safety profile, particularly in reducing bleeding risks. However, as an observational study, it lacks the randomization and control of an RCT, which limits its ability to establish causality. This study partially fills the gap in understanding NOAC-specific efficacy and safety but should be validated by prospective trials [44].

The RIVER trial is pivotal among RCTs, showing that rivaroxaban is non-inferior to warfarin in AF patients with a bioprosthetic mitral valve. While this RCT provides high-quality evidence, it does not demonstrate the superiority of rivaroxaban over warfarin, leaving some uncertainty regarding optimal anticoagulation in this population [47].

Similarly, the ARISTOTLE trial reinforces the efficacy and safety of apixaban in AF patients with prior bioprosthetic valve replacement or repair, making it an earlier landmark study supporting NOAC use in valvular settings. However, the ARISTOTLE trial focused on a specific subgroup rather than evaluating NOAC use across a broader VHD population [47].

While NOACs have revolutionized anticoagulation therapy for AF, their use in patients with moderate to severe MS remains contraindicated due to a lack of robust evidence and specific mechanistic concerns [47,48]. Warfarin continues to be the preferred option for this high-risk population [48]. In contrast, NOACs are a viable alternative for patients with Type 2 VHD, with apixaban emerging as a particularly effective and safe option [48]. Future research should focus on direct comparisons between NOACs and prospective trials to refine anticoagulation strategies for patients with valvular AF.

Table 3 shows the characteristic features of different comparative studies based on different anticoagulation responses.

**Table 3:** Comparative analysis of different studies based on different anticoagulants NOACs: novel oral anticoagulants, SE: systemic embolism, AF: atrial fibrillation, VHD: valvular heart disease, RR: relative risk, CI: confidence interval, VKAs: vitamin K antagonists, RCT: randomized controlled trial, HR: hazard ratio, VTE: venous thromboembolism, OR: odds ratio, DOACs: direct oral anticoagulants

Author	Year	Type of study	Population (cases vs. controls)	Statistical results	Comments
Liang et al. [41]	2024	Meta-analysis	16,070 patients with AF and type 2 VHD	RR for stroke/SE: 0.75 (95% CI: 0.64-0.89, p=0.0005); RR for major bleeding: 0.88 (95% CI: 0.64-1.21, p=0.43)	NOACs reduce stroke/SE risk and have comparable significant bleeding risk to VKAs link
Yoon et al. [42]	2023	Real-world data emulation	Emulated RCTs using the South Korean nationwide claims database	AMPLIFY: RR 0.81 (95% CI: 0.70-0.94); RE-COVER II: HR 0.60 (95% CI: 0.37-0.96); Hokusai-VTE: HR 0.49 (95% CI: 0.31-0.78)	Real-world data supports the effectiveness of NOACs in VTE link
Adhikari et al. [43]	2021	Systematic review and meta-analysis	Patients with AF and bioprosthetic valves	OR for thromboembolism: 0.72 (95% CI: 0.44-1.17); OR for major bleeding: 0.66 (95% CI: 0.48-0.89, p=0.006)	DOACs might decrease the risk of significant bleeding without increasing the risk of thromboembolism
Dawwas et al. [44]	2022	Population-based study	Patients with AF and VHD	HR for ischemic stroke/systemic embolism: 0.57 (95% CI: 0.40-0.80); HR for major bleeding: 0.67 (95% CI: 0.63-0.72)	Apixaban shows superior effectiveness and safety compared to rivaroxaban link
Guimarães et al. [47]	2020	RCT	1,005 patients with AF and bioprosthetic mitral valve	HR for stroke: 0.25 (95% CI: 0.07-0.88); HR for major bleeding: 0.54 (95% CI: 0.21-1.35)	Rivaroxaban is non-inferior to warfarin for preventing thromboembolic events link
Guimarães et al. [48]	2019	RCT	Patients with AF and prior bioprosthetic valve replacement or repair	HR for stroke/systemic embolism: 0.64 (95% CI: 0.59-0.70); HR for major bleeding: 0.67 (95% CI: 0.63-0.72)	Apixaban is effective and safe in patients with bioprosthetic valves link

Scope for future studies and clinical trials

Numerous studies have demonstrated the efficacy of VKAs in managing AF in patients with specific types of VHD or valve replacements [31]. However, the limited application of NOACs in patients with AF and VHD, particularly in high-risk groups, is primarily due to the lack of robust clinical trials exploring their use in these populations [32,33].

To address this gap, future clinical trials should focus on evaluating a single type of NOAC in patients with AF and a specific type of VHD. Ensuring rigorous study designs and large sample sizes will be essential to yield reliable and generalizable results [33-35]. Based on existing data gaps, certain NOAC-VHD combinations should be prioritized for future research [33].

For example, apixaban in MS could be explored further. Given the contraindication of NOACs in moderate to severe MS, studies could assess the safety and efficacy of apixaban in mild MS or post-valvuloplasty patients, where the thrombotic risk may be lower [33,34]. Similarly, edoxaban has shown promise in AF patients with bioprosthetic valves, but further trials are needed to confirm its efficacy and safety compared to VKAs [33].

Rivaroxaban could be studied in patients with aortic stenosis or regurgitation, particularly in those with concurrent AF, to assess its thromboembolic and bleeding risks. Additionally, dabigatran’s role in AF patients with tricuspid regurgitation or stenosis remains underexplored and warrants further investigation [35]. These targeted studies would provide valuable insights into the optimal use of NOACs in specific VHD populations, addressing current limitations in the evidence base [31-35].

Conducting trials in high-risk VHD patients presents significant ethical and logistical challenges [31]. High-risk patients, such as those with mechanical heart valves or severe MS, are more prone to thromboembolic and bleeding complications, raising concerns about patient safety [35]. The RE-ALIGN trial, which evaluated dabigatran in patients with mechanical heart valves, was terminated early due to increased rates of stroke and bleeding, highlighting the potential risks of NOACs in this population [67]. As a result, future studies may need to exclude mechanical valve patients entirely or focus on lower-risk subgroups, such as those with bioprosthetic valves or mild VHD, to ensure patient safety [67].

Genetic variations and drug interactions play a critical role in the metabolism and efficacy of both NOACs and VKAs [65]. For NOACs, polymorphisms in genes encoding drug transporters (e.g., P-glycoprotein (P-gp)) and metabolizing enzymes (e.g., CYP3A4) can significantly affect drug levels and response [36]. Patients with reduced CYP3A4 activity may have higher plasma concentrations of NOACs like rivaroxaban and apixaban, increasing bleeding risks [37]. Furthermore, drugs that inhibit or induce P-gp (e.g., verapamil, rifampin) can alter NOAC absorption and clearance, necessitating dose adjustments or alternative therapies [38].

In contrast, VKAs are affected by polymorphisms in the VKORC1 and CYP2C9 genes, which influence warfarin dosing and stability [36]. While both NOACs and VKAs are susceptible to drug interactions, NOACs may offer advantages in patients with complex medication regimens due to their fewer dietary restrictions and more predictable pharmacokinetics [37].

Large-scale RCTs with long follow-up periods are needed to assess outcomes such as thromboembolism, bleeding, and mortality in specific VHD populations [38]. Registry-based studies and propensity score-matched analyses can complement RCTs by providing real-world evidence on NOAC use in diverse patient populations. These studies are particularly valuable for assessing long-term outcomes and rare adverse events [38-40].

Adaptive designs allow for modifications to the trial protocol based on interim results, improving efficiency and patient safety [41]. For example, trials could initially include a broad population and then narrow the focus to specific subgroups based on early findings [41,42].

While VKAs remain the standard of care for many patients with AF and VHD, NOACs offer a promising alternative for specific subgroups [38-42]. Future studies should focus on targeted NOAC-VHD combinations, employ robust trial designs, and address ethical and logistical challenges to ensure patient safety. By generating high-quality evidence, these studies can inform new protocols and guidelines for the safe and effective use of NOACs in high-risk VHD populations.

## Conclusions

Managing concurrent VHD and AF, particularly with anticoagulant therapy, presents nuanced challenges. The primary goal of treatment is to balance the reduction of thromboembolism and bleeding risks while considering specific VHD types and individual patient factors. Among different VHDs, MS and AVH significantly increase thromboembolism and bleeding risks, regardless of AF and other comorbidities. Therefore, careful consideration is required before altering existing guidelines or introducing new drugs, even in clinical trials. The latest AHA/ACC guidelines recommend a collaborative decision-making approach based on CHA2DS2-VASc scores for OAC in patients with AF and VHD, excluding high-risk VHDs such as MS and mechanical bioprosthetic valves. Studies generally favor NOACs over warfarin, demonstrating positive outcomes in reducing SEE, MI, and ICH. However, the EHRA provides a more nuanced classification, allowing NOAC use in certain VHD subtypes while restricting it in others. For example, NOACs are recommended in patients with Type 2 VHD (e.g., non-rheumatic mitral valve disease, aortic valve disease) and bioprosthetic valves after the initial three-month postoperative period. At the same time, they remain contraindicated in moderate to severe MS and mechanical valves due to insufficient supporting evidence.

The disparity in NOAC use for patients with AVH and MS stems from differences in clinical outcomes, regulatory approvals, and physician preferences. Limited studies and trials using NOACs in patients with AF and VHD have produced varied results depending on the specific NOAC, its pharmacological properties, and patient factors. For instance, the RE-ALIGN study on dabigatran in mechanical AVH patients was discontinued due to increased stroke and bleeding risks, highlighting concerns about suboptimal anticoagulation levels and mechanical valves' heightened thrombotic risk. These findings raise ethical and safety concerns about testing NOACs in high-risk groups and suggest that future trials should exclude dabigatran or explore alternative dosing strategies. While limited data exist on NOACs in MS patients, the lack of evidence supporting their superiority over warfarin justifies the continuation of current guidelines. However, the potential benefits of NOACs over VKAs could warrant well-designed clinical trials assessing their efficacy and safety, even in high-risk patients. Given VHD's heterogeneity, future research should focus on individualized anticoagulation strategies rather than universally applying a single NOAC, identifying the safest and most effective options for specific VHD subtypes based on patient-specific factors such as renal function, age, and comorbidities.
